# Rare Species Shift the Structure of Bacterial Communities Across *Sphagnum* Compartments in a Subalpine Peatland

**DOI:** 10.3389/fmicb.2019.03138

**Published:** 2020-01-21

**Authors:** Wen Tian, Xing Xiang, Liyuan Ma, Stephanie Evers, Ruicheng Wang, Xuan Qiu, Hongmei Wang

**Affiliations:** ^1^State Key Laboratory of Biogeology and Environmental Geology, China University of Geosciences, Wuhan, China; ^2^School of Environmental Studies, China University of Geosciences, Wuhan, China; ^3^School of Natural Sciences and Psychology, Liverpool John Moores University, Liverpool, United Kingdom; ^4^TROCARI (Tropical Catchment Research Initiative), Semenyih, Malaysia; ^5^Laboratory of Basin Hydrology and Wetland Eco-Restoration, China University of Geosciences, Wuhan, China

**Keywords:** *S. palustre*, bacterial communities, rare species, compartment effects, co-occurrence network, the Dajiuhu Peatland

## Abstract

*Sphagnum*-associated microbiomes are crucial to *Sphagnum* growth and peatland ecological functions. However, roles of rare species in bacterial communities across *Sphagnum* compartments are poorly understood. Here the structures of rare taxa (RT) and conditionally abundant and rare taxa (CART) from *Sphagnum palustre* peat (SP), *S. palustre* ectosphere (Ecto) and *S. palustre* endosphere (Endo) were investigated in the Dajiuhu Peatland, central China. Our results showed that plant compartment effects significantly altered the diversities and structures of bacterial communities. The Observed species and Simpson indices of RT and CART in alpha diversity significantly increased from Endo to SP, with those of Ecto in-between. The variations of community dissimilarities of RT and CART among compartments were consistent with those of whole bacterial communities (WBC). Network analysis indicated a non-random co-occurrence pattern of WBC and all keystone species are affiliated with RT and CART, indicating their important role in sustaining the WBC. Furthermore, the community structures of RT and CART in SP were significantly shaped by water table and total nitrogen content, which coincided with the correlations between WBC and environmental factors. Collectively, our results for the first time confirm the importance of rare species to bacterial communities through structural and predicted functional analyses, which expands our understanding of rare species in *Sphagnum*-associated microbial communities in subalpine peatlands.

## Introduction

Peatlands are the largest terrestrial carbon pool and important nitrogen reservoir, covering an area of 4 × 10^6^ km^2^ on the Earth (Limpens et al., 2008). Globally, northern peatlands store 473 ∼ 621 Gt C and 8 ∼ 15 Gt N, which approximately account for 33% of the soil organic C and 9 ∼ 16% of the terrestrial N (Limpens et al., 2006; Yu et al., 2010), respectively. These abundant organic matters mainly originate from *Sphagnum* mosses compiling about 300 species around the world (Daniels, 1990; Shaw et al., 2003). *Sphagnum* mosses are the prevalent vegetation in acidic peatlands and greatly contribute to peatland development. *Sphagnum* mosses host diverse bacterial communities either endosymbiotic or ectosymbiotic with the plant (Opelt et al., 2007a, b; Bragina et al., 2012, 2014; Tian et al., 2019). The bacterial communities can produce antifungal compounds to improve pathogen defense of hosts and provide nitrogen for *Sphagnum* mosses via nitrogen-fixing bacteria such as *Burkholderia* (Opelt et al., 2007c; Shcherbakov et al., 2013, 2015). Besides the above-mentioned functions, endosymbiotic methanotrophs are demonstrated to provide carbon dioxide for the photosynthesis of *Sphagnum* mosses via methane oxidation (Raghoebarsing et al., 2005; Larmola et al., 2010; Kip et al., 2011). Therefore these *Sphagnum*-associated microorganisms play important roles in *Sphagnum* growth and elemental cycling in peatland ecosystems (Bragina et al., 2014; Weston et al., 2015; Kostka et al., 2016).

Generally, microbial communities in peatlands are comprised of a few dominant taxa (relative abundance >1%) with a long tail of low abundance species (Gilbert and Mitchell, 2006; Andersen et al., 2013; Tian et al., 2019), as well as in other habitats (Delgado-Baquerizo et al., 2018). These abundant taxa largely contribute to carbon cycling and energy flow due to their broad niche, high competitive ability and fast growth rate (Campbell et al., 2011). However, little knowledge is known about the low abundance populations, designated as rare biosphere (Sogin et al., 2006), or opportunistic species (Jousset et al., 2017), with high diversity. They may be part of the microbial “seed bank” and contribute disproportionately to community diversities and variations (Pedrós-Alió, 2007; Shade et al., 2014). They may become dominant and conduct essential functions in nutrient cycling, which compensate for the function deficiency of abundant species (Jousset et al., 2017) under favorable environmental conditions (Pedrós-Alió, 2007; Aanderud et al., 2015). Additionally, the responses of abundant and rare communities to environmental factors are different (Chen et al., 2017; Ruiz-González et al., 2019) due to the differences in niche breadth, competitiveness of resources and growth rate (Wu et al., 2017; Xue et al., 2018). Previous studies about *Sphagnum*-associated microbial communities mainly concentrate on abundant taxa, whereas the distribution of rare species among different compartments of *Sphagnum* (surface peat, ectosphere and endosphere of *Sphagnum*) still remains largely unknown. Therefore, we hypothesize that (1) rare species are major contributors to bacterial community variations across different compartments of *Sphagnum palustre*; (2) rare species may play an important role in the whole co-occurrence network and potential functions of bacterial communities; (3) rare species and abundant taxa may response differently to environmental factors in surface peat.

To test our hypotheses, bacterial communities were investigated via 16S rRNA gene sequencing from different compartments of *S. palustre* (*S. palustre* peat, *S. palustre* ectosphere, and *S. palustre* endosphere) in the Dajiuhu Peatland, Central China. In present study, we aim to address the following questions: (1) compare the diversity and composition of abundant and rare species, and their relative contributions to the variation of whole bacterial communities; (2) reveal the co-occurrence patterns and potential functional variations of abundant and rare species in *Sphagnum* compartments; (3) explore the response in abundant and rare species to environmental factors in *S. palustre* peat. Altogether, this study will enhance our understanding of *Sphagnum*-associated bacterial communities in different compartments, with emphasis on rare species.

## Materials and Methods

### Depiction of Site and Sampling

Dajiuhu (31°24′ – 31°33′ N, 109°56′ – 110°11′ E) is a typical subalpine peatland located in Shennongjia, Hubei province, central China (Supplementary Figure S1A). Peat has developed in a closed intermontane basin (1,730 m, above sea level), with an area around 16 km^2^ and a depth of 2–3 m (Huang et al., 2018). The mean annual temperature and precipitation are 7.2°C and 1,560 mm, respectively. The present vegetation types are mainly *Carex* spp., *S. palustre*, *Sanguisorba officinalis*, and *Euphorbia esula* accompanying shrubs. More information about the Dajiuhu Peatland was given by Huang et al. (2012) and Li et al. (2016).

In this study, *S. palustre* and underlying peat samples (0–5 cm) were collected with sterile plastic bags and centrifuge tubes, respectively. Samples were collected in triplicate, in July 2016. Sampling sites were located at the peatland core area are Erhaoba (EHB), Niangniangfen (NNF) and two sites at Yangluchang (YLC) where *S. palustre* was actively growing (Supplementary Figure S1A). Water table at the four sites was determined *in situ* at the time of sampling. Visible plant root, litter and debris were removed from *S. palustre* peat samples. All the samples were transported to the geomicrobiology laboratory at China University of Geosciences (Wuhan) with ice box within 12 h.

### Sample Processing

To get the microbes from the ectosphere, *S. palustre* was rinsed three times for 5 min each time with sterile deionized water to remove adherent debris. Then approx. 5 g of *S. palustre* samples were placed in 20 mL sterile phosphate buffered saline (PBS), and shaken and sonicated to separate the tightly adhering microbes (Bulgarelli et al., 2015; Edwards et al., 2015). The *S. palustre* plant material was transferred into another tube, and PBS solution was centrifuged at 10,000 *g* for 5 min and the supernatant was discarded (Supplementary Figure S1B). The remaining PBS fraction was stored at −80°C until DNA extraction.

The retrieved *S. palustre* designated for endosphere underwent further sonication and shake twice following the above-depicted approach. The *S. palustre* samples were then placed into 75% ethanol and 20% hydrogen peroxide for 1 min, respectively (Bulgari et al., 2009). The *S. palustre* samples were rinsed three times for 5 min each time with sterile deionized water and the final sterile water was inoculated on the R2A medium. The *S. palustre* plant materials were stored at −80°C until DNA extraction.

Additional *S. palustre* peat samples (approx. 20 g) were dried at 50°C for 24 h and subject to the organic matter and total nitrogen analyses.

### DNA Extraction, Amplification, and Sequencing

Total DNA for each approximately 0.5 g freeze-dried *S. palustre* peat, ectosphere and endosphere fractions were extracted by PowerSoil DNA Isolation Kit (QIAGEN, Düsseldorf, Germany). The concentration and quality of DNA were checked with NanoDrop 2000 spectrophotometer (Thermo Fisher Scientific, Waltham, MA, United States) and 1% agarose gel stained with ethidium bromide. The V3-V4 regions of bacterial 16S rRNA gene were amplified with the primer set of 347F (5′-CCTACGGRRBGCASCAGKVRVGAAT-3′) and 802R (5′-GGACTACNVGGGTWTCTAATCC-3′) (Ren et al., 2019). PCR reactions contained 25 μL 2× Premix Taq (Takara Biotechnology, Dalian Co., Ltd., China), 1 μL each primer (10 mM) and 3 μL DNA (20 ng/μL) template in a volume of 50 μL. PCR products were then purified and quantified with Quit 2.0 fluorometer (Invitrogen, Carlsbad, CA, United States). DNA libraries were generated using NEBNext Ultra DNA Library Prep Kit for Illumina (New England Biolabs, Ipswich, MA, United States) following manufacturer’s protocols and the quality of libraries was validated by the Agilent 2100 Bioanalyzer (Agilent Technologies, Palo Alto, CA, United States) and the Qubit 2.0 Fluorometer. DNA libraries were sequenced on an Illumina MiSeq platform with PE300 paired-end reads at GENEWIZ, Inc. (Suzhou, China).

### Sequence Analysis

Raw sequences were processed in Quantitative Insight Into Microbial Ecology (QIIME v. 1.9.1) platform (Caporaso et al., 2010). The sequences were assigned to each sample based on their unique barcode, followed by the removal of primers and barcodes. The forward and reverse sequences were paired-end assembly with overlaps more than 20 bp. The high quality reads were determined according to without any ambiguous base “N”, length >200 bp and base quality score >20. The clean sequences were clustered into operational taxonomic units (OTUs) by UCLUST (Edgar, 2010) based on 3% sequence dissimilarity and singletons (OTU with only one sequence in all samples) were eliminated. The mitochondria and chloroplast were removed and the chimera sequences were identified by UCHIME algorithm (Edgar et al., 2011). Represent sequences for each OTU were classified using Ribosomal Database Project classifier at a threshold of 0.8 (Cole et al., 2009) and annotated in the SILVA 119 database. All the samples were resampled to 29,577 sequences per sample by mothur (Schloss et al., 2009).

Raw 16S rRNA gene data was available at the NCBI Sequence Read Archive database^1^ with the accession number of PRJNA512496.

### Definition of OTU Classification

We set relative abundance thresholds as 0.01% for rare taxa (RT) and 1% for abundant taxa (AT) and classified all OTUs (archaea and unclassified OTUs excluded) into six subcategories (Dai et al., 2016; Xue et al., 2018) to compare the roles of taxa with different abundance in the whole bacterial communities (WBC): always abundant taxa (AAT) with a relative abundance >1% in all samples; conditionally abundant taxa (CAT) with a relative abundance ≥1% in some samples and always ≥0.01% in all samples; always rare taxa (ART) with a relative abundance <0.01% in all samples; conditionally rare taxa (CRT) with a relative abundance <0.01% in some samples but never abundant (≥1%) in any samples; moderate taxa (MT) with a relative abundance between 0.01% and 1% in all samples; conditionally abundant and rare taxa (CART) with a relative abundance varying from rare (<0.01%) to abundant (≥1%). The detailed depictions of OTU category were listed in Supplementary Table S1.

### Date Processing and Analysis

The contribution of each subcategory to community variations was determined by similarity percentage (SIMPER) analysis in PAST 3.26^2^. The alpha diversity indices (Observed species, Chao1, ACE, Simpson, Pielou, and Shannon), NMDS, PERMANOVA, and ANOSIM analyses were performed using “vegan” package in R 3.5.1^3^. Functional profiles were predicted using Tax4Fun package (Aßhauer et al., 2015) and principal component analysis (PCA) was employed to reveal compartment differences of potential functional communities based on Euclidean distance of the relative abundance of overall Kyoto Encyclopedia of Genes and Genomes (KEGG) Orthologs (KOs) with “ggord” package in R 3.5.1. Differences in alpha diversity and potential function were tested with one-way analysis of variance (ANOVA) in SPSS 18^4^. Beta diversity analysis was performed based on Bray-Curtis distance of the relative abundance of OTUs in non-metric multidimensional scaling (NMDS). Analysis of similarity (ANOSIM) was used to test the differences among groups. Monte–Carlo tests of redundancy analysis determined the relationships between environmental factors and bacterial communities of *S. palustre* peat in Canoco 5^5^.

The OTUs with relative abundance above 0.01% were selected, and the correlation matrix between two OTUs was calculated in “psych” R package. Correlation coefficient (*r* > 0.8 or *r* < −0.8) and significance (FDR-corrected *P* < 0.01) were integrated into network analysis (Jiao et al., 2016). Topology attributes of the network (numbers of node and edge, average degree, network diameter, graph density, modularity, clustering coefficient and average of path length) were calculated using “igraph” package in R 3.5.1. Network visualization was conducted with Gephi 0.9.2^6^. Meanwhile, 1,000 Erdös-Réyni random networks were constructed to compare with the real network (Erdös and Réyni, 1960). Nodes with high degree (>100) and low betweenness centrality (<5,000) were considered as the keystone species in co-occurrence networks (Ma et al., 2016).

## Results

### Diversity of Bacterial Communities

In total, 2,413,649 high quality sequences and 3,144 operational taxonomic units (OTUs) were obtained. After the removal of archaea and unclassified OTUs, 2,848 OTUs were subject to further analysis. Among these, 13 OTUs representing 9.01% of all sequences were abundant taxa (AT) and present in all samples, whereas 2,706 OTUs occupying 39.2% of all sequences belonged to rare taxa (RT). Conditionally abundant and rare taxa (CART) included 126 OTUs representing 51.13% of all sequences (Supplementary Table S1).

**FIGURE 1:**
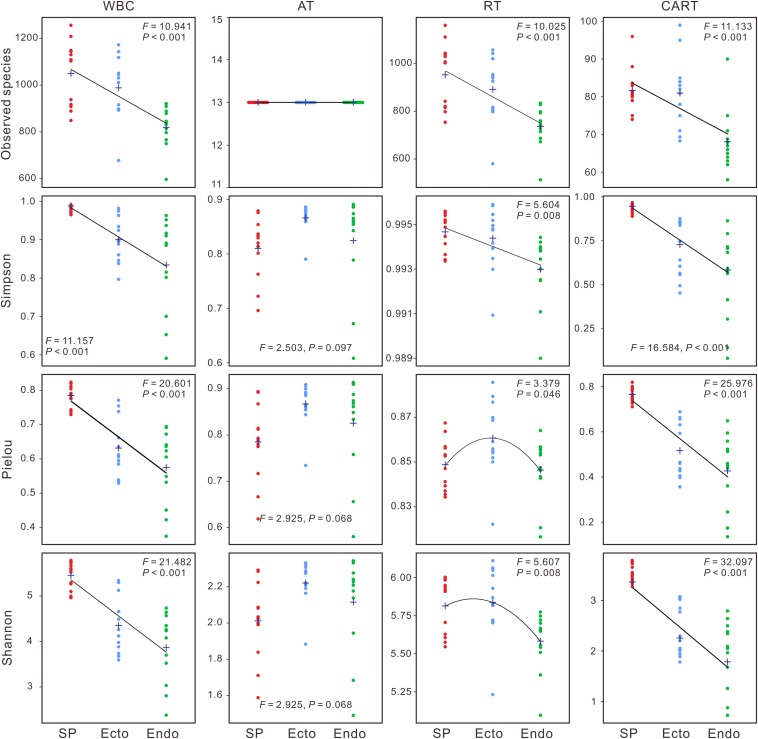
Changes in alpha diversity (Observed species, Simpson, Pielou, and Shannon indices) across *Sphagnum palustre* compartments. One-way analysis of variance (ANOVA) is used to compare the differences among *S. palustre* compartments. Cross sign represents the mean value (*n* = 12). WBC, whole bacterial communities; AT, abundant taxa; RT, rare taxa; CART, conditionally abundant and rare taxa; SP, *S. palustre* peat; Ecto, *S. palustre* ectosphere; Endo, *S. palustre* endosphere. The *F* and *P*-values of Observed species cannot be calculated in AT. Colored dots represent SP, Ecto, and Endo samples, respectively. Lines are fitted curves.

**FIGURE 2:**
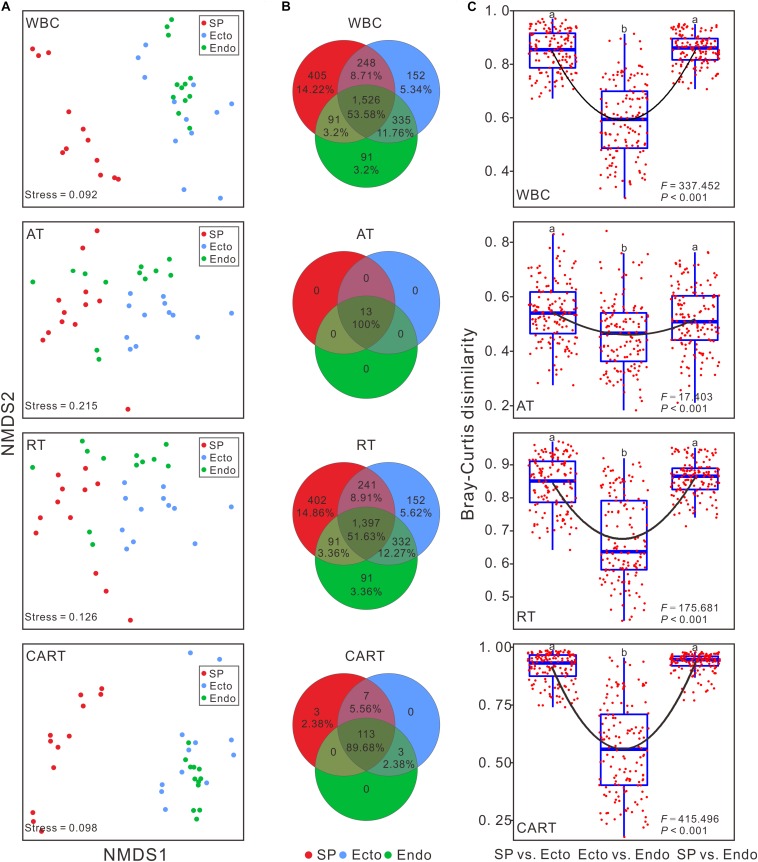
Structural variations of bacterial communities among *S. palustre* compartments. **(A)** Structure of bacterial communities based on Bray-Curtis distance in NMDS. **(B)** The number of shared and unique OTUs across different compartments of *S. palustre*. **(C)** The community dissimilarities of bacterial communities between *S. palustre* compartments. Each box indicates the maximum, minimum, 75th and 25th quartiles, respectively, and line within each box represents the median (*n* = 144). Lowercase letters indicate significances at the 95% confidence interval according to ANOVA with Tukey’s HSD *post hoc* test. The abbreviations are the same as indicated in Figure 1.

Significant differences were observed in alpha diversities of whole bacterial communities (WBC) and RT among compartments (Supplementary Table S2). Observed species, Simpson, Pielou, and Shannon indices of WBC and CART significantly decreased from *Sphagnum palustre* peat (SP) to *S. palustre* endosphere (Endo), with those of *S. palustre* ectosphere (Ecto) in-between (Figure 1). In contrast, there were no significant differences in alpha diversity across sampling sites (Supplementary Table S2). Venn diagrams indicated over 50% OTUs in WBC were shared in three compartments, and the distributions of shared and specific OTUs in RT were similar to those of WBC (Figure 2B).

The dissimilarities in bacterial communities across compartments were visualized by NMDS analysis based on Bray-Curtis matrix. The communities of the same compartment clustered together (Figure 2A). The pairwise comparison of between group distances (Figure 2C), and the results of ANOSIM (Table 1) indicated that bacterial communities were significantly different among compartments, rather than among sampling sites.

**TABLE 1:** Dissimilarity test showing differences of bacterial communities across compartments and sampling sites.

	**ANOSIM**							
	**WBC**		**AT**		**RT**		**CART**	
	***R***	***P***	***R***	***P***	***R***	***P***	***R***	***P***
**Compartment**								
SP vs. Ecto	0.837	**0.001**	0.641	**0.001**	0.750	**0.001**	0.869	**0.001**
SP vs. Endo	0.919	**0.001**	0.431	**0.001**	0.765	**0.001**	0.972	**0.001**
Ecto vs. Endo	0.167	**0.009**	0.308	**0.002**	0.178	**0.015**	0.135	**0.009**
**Sampling site**								
E1 vs. N1	0.029	0.238	0.003	0.361	0.081	0.167	0.029	0.243
E1 vs. Y2	0.004	0.346	0.019	0.310	0.075	0.164	−0.024	0.564
E1 vs. Y6	0.306	**0.007**	0.019	0.310	0.642	**0.002**	0.245	**0.018**
N1 vs. Y2	0.035	0.210	0.082	0.142	0.137	0.111	0.010	0.300
N1 vs. Y6	0.306	**0.007**	0.436	**0.001**	0.533	**0.001**	0.247	**0.012**
Y2 vs. Y6	0.327	**0.003**	0.371	**0.005**	0.481	**0.002**	0.224	**0.027**

*ANOSIM, analysis of similarity; SP, *Sphagnum palustre* peat; Ecto, *S. palustre* ectosphere; Endo, *S. palustre* endosphere; E1, the first site of Erhaoba; N1, the first site of Niangniangfen; Y2, the second site of Yangluchang; Y6, the sixth site of Yangluchang. WBC, whole bacterial communities; AT, abundant taxa; RT, rare taxa; CART, conditionally abundant and rare taxa. Dissimilarities of bacterial communities are calculated based on Bray-Curtis distance. *P*-values are corrected by false discovery rate (FDR) in BH method. Bold font represents significant values (α = 0.05).*

### Composition of Bacterial Communities

**FIGURE 3:**
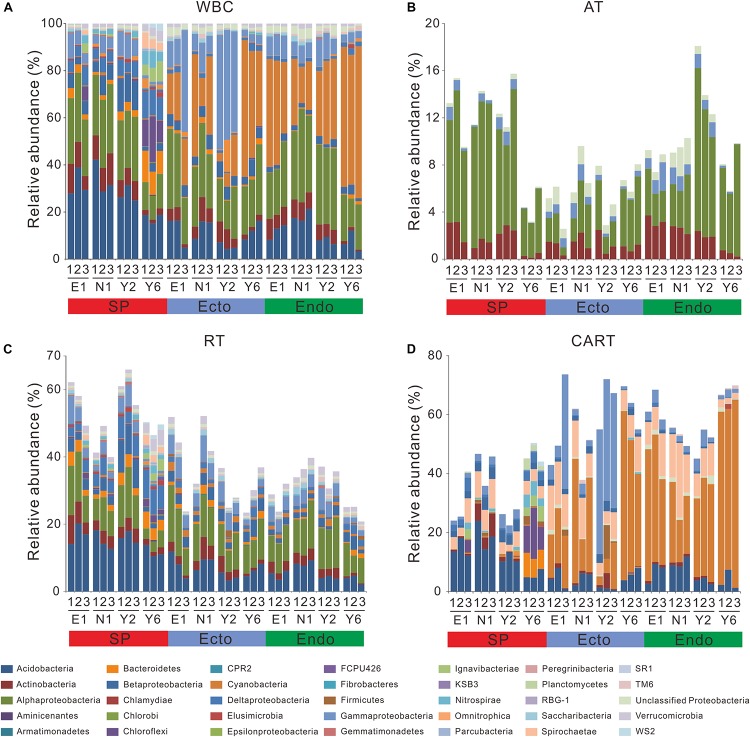
Composition of bacterial communities in WBC **(A)**, AT **(B)**, RT **(C)**, and CART **(D)**. E1, the first site of Erhaoba; N1, the first site of Niangniangfen; Y2, the second site of Yangluchang; Y6, the sixth site of Yangluchang. Others abbreviations are described in Figure 1.

As for WBC, Acidobacteria and Alphaproteobacteria dominated SP, whereas the predominant phyla in Ecto were Cyanobacteria, Alphaproteobacteria, and Gammaproteobacteria, followed by Acidobacteria. In Endo, Cyanobacteria and Alphaproteobacteria dominated the communities, followed by Acidobacteria (Figure 3A). Alphaproteobacteria was commonly found across all compartments and Acidobacteria presented frequently in SP, whereas Cyanobacteria was abundant in Ecto and Endo.

In total, 13 OTUs were classified into AT, which were affiliated to Actinobacteria, Alphaproteobacteria and Gammaproteobacteria phyla (Figure 3B), and accounted for 0.46% of the OTU richness (Supplementary Table S3). As many as 2,706 OTUs were identified as RT, which were affiliated with 30 phyla (Figure 3C) and represented 95.01% of the OTU richness (Supplementary Table S3). Furthermore, 126 OTUs were recognized as CART belonging to 13 phyla (Figure 3D), and represented 4.42% of the OTU richness (Supplementary Table S3).

### Co-occurrence Network

**FIGURE 4:**
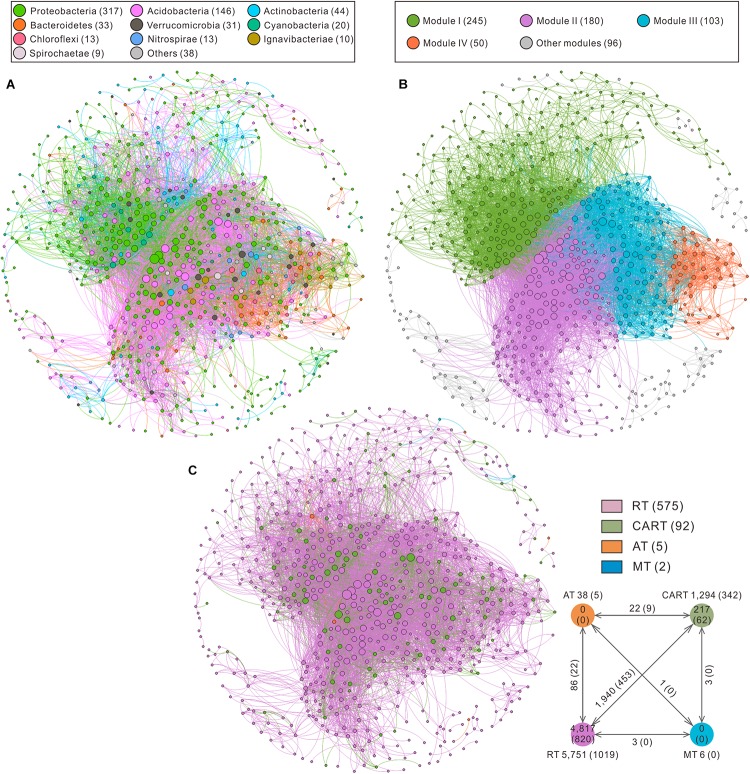
Co-occurrence network of bacterial communities sorted in color by phylum **(A)**, modularity **(B)** and OTU category **(C)** rank, respectively. A connection between two nodes stands for a significant correlation (*r* > 0.8 or *r* < –0.8, *P* < 0.01). The numbers inside parentheses indicate the number of nodes in **(A,B,C)**. Each node represents a single OTU. Positive edge and negative edge numbers among taxa with various abundances are put outside and inside the parentheses in a small panel at the bottom right corner. The size of each node is proportional to its degree; the thickness of edge linking two nodes is proportional to the corresponding correlation coefficient. RT, rare taxa; CART, conditionally abundant and rare taxa; AT, abundant taxa; MT, moderate taxa.

A co-occurrence network was constructed based on correlation relationships among OTU relative abundances. The resulting network contained 674 nodes linked by 8,455 edges, where positive correlations were commonly observed compared to negative ones (Figure 4C). The scale-free degree distribution of the real network (power-law: *R*^2^ = 0.945, *P* < 0.0001, Supplementary Figure S2) indicated a scale-free and non-random network structure. Meanwhile the network had small-world features and modular structure as indicated by higher values of the modularity index (0.762) and clustering coefficient (0.521) compared with those of identical size Erdös-Réyni random networks (Erdös and Réyni, 1960; Table 2).

**TABLE 2:** Topological properties of co-occurrence networks of microbial communities in the Dajiuhu Peatland.

	**Average degree**	**Network diameter**	**Graph density**	**Modularity**	**Clustering coefficient**	**Average path length**
Real network	25.089	12	0.037	0.762	0.521	3.442
Random network	6.720 (0.138)	6.779 (0.498)	0.0100 (0.0002)	0.302 (0.010)	0.0099 (0.0014)	3.630 (0.035)

*Random network is an identically sized random network generated by wiring all the links with the same numbers of nodes and edges to the real network. The topological features are calculated as the average value of 1,000 Erdös-Réyni random networks. The values in parentheses are standard deviations.*

The top 10 phyla in the network accounted for 93% of all nodes (Figure 4A). The whole network was grouped into 4 major modules (Figure 4B). Module I included 36.35% of the nodes in the entire network, which mainly fell into Proteobacteria. Module II and module III were mainly comprised of Proteobacteria and Acidobacteria occupying 41.99% of the nodes in the whole network, whereas module IV mainly consisted of Proteobacteria and Bacteroidetes (Supplementary Figure S3A). Ternary plots showed the specificity of different modules to a particular compartment (Supplementary Figures S3B–E). For example, most OTUs in module I showed high abundances in Ecto and Endo, whereas the majority of OTUs in modules II, III, and IV showed high proportions in SP.

Node-level topological features of microbial groups with different abundances (RT, CART, AT, and MT) indicated that the degree, betweenness and eigenvector centrality values of CART were significantly higher than that of RT (Supplementary Figures S4A,B,D). The closeness centrality values of AT were the highest among 4 microbial groups (Supplementary Figure S4C). Based on high degree (>100) and low betweenness centrality (<5,000) in the co-occurrence networks, 5 OTUs were identified as keystone species including Acidobacteria (1 OTU), Betaproteobacteria (1 OTU), Alphaproteobacteria (1 OTU), Ignavibacteriae (1 OTU) and Spirochaetae (1 OTU), which belonged to RT and CART (Supplementary Table S4).

### Environmental Factors Related to Bacterial Communities

Monte–Carlo tests indicated that three physicochemical parameters, i.e., water table, total nitrogen and organic matter contents were significantly correlated with the variations of WBC, RT, CART, and AT in SP samples (Table 3). The WBC was significantly affected by water table and total nitrogen content (*P* < 0.05), which were also identified as the significant determinants (*P* < 0.05) in RT and CART. In contrast, organic matter content rather than water table and total nitrogen significantly (*P* < 0.05) shaped the structure of AT.

**TABLE 3:** Correlations between bacterial communities and environmental factors in *S. palustre* peat samples in the Dajiuhu Peatland.

	**WBC**			**AT**			**RT**			**CART**		
	**Explanation of variation (%)**	***F***	***P***	**Explanation of variation (%)**	***F***	***P***	**Explanation of variation (%)**	***F***	***P***	**Explanation of variation (%)**	***F***	***P***
WT	32.3	4.8	**0.025**	36.8	5.8	0.060	30.6	4.4	**0.015**	42.1	7.3	**0.010**
TN	24.6	5.1	**0.010**	2.1	1.0	0.499	24.3	4.8	**0.010**	26.0	7.4	**0.010**
OM	7.4	1.7	0.203	38.1	13.7	**0.010**	3.8	0.9	0.544	5.7	1.8	0.210

*Correlations between bacterial communities and environmental factors are determined by Monte–Carlo tests of redundancy analysis in Canoco 5. WT, water table; TN, total nitrogen; OM, organic matter. *P*-values are adjusted through multiple comparisons using FDR. Bold font represents significant values (α = 0.05). Other abbreviations are the same as indicated in Table 1.*

### Potential Functions of Bacterial Communities

Similar to the structural variations of communities, the potential functions of communities showed an obvious separation among WBC, RT, and CART (Supplementary Figures S5A,C,D) compared to that in AT across compartments (Supplementary Figure S5B) as indicated by principal component analysis (PCA) based on Euclidean distance. The dissimilarity comparisons in each two compartments and sampling sites were further quantified using PERMANOVA (Supplementary Table S5), which showed significant (α = 0.05) differences in functional potentials across compartments rather than those among sampling sites.

**FIGURE 5:**
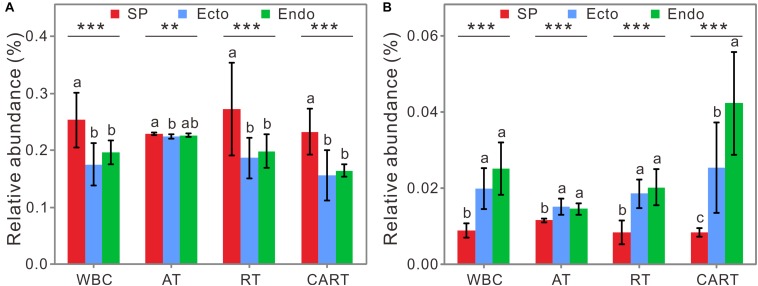
The relative abundances of predicted nitrogen-fixing **(A)** and methane-oxidizing groups **(B)** in WBC, AT, RT, and CART. Abbreviations are the same as those in Figure 1. Significant differences (ANOVA tests) are labeled as follows: ^∗∗^*P* < 0.01; ^∗∗∗^*P* < 0.001. Lowercase letters represent significant differences at 95% confidence interval.

Among WBC, RT, and CART, the relative abundance of potential nitrogen-fixing functional groups was the highest in SP, followed by those in Endo, and the lowest was in Ecto (Figure 5A). The relative abundance of predicted methane-oxidizing functional groups significantly decreased from Endo to SP, with that of Ecto in-between (Figure 5B). Together, specific potential functional groups such as N_2_-fixers and CH_4_-oxidizers in RT and CART can reflect consistent variation patterns with those in WBC across compartments, whereas AT did not.

## Discussion

The main objective of this study was to explore the roles of taxa with various abundances on the shift of bacterial communities in *Sphagnum* compartments at a subalpine peatland. Our results revealed that rare species (referring to RT and CART and hereafter) were the major contributors to the shift of *Sphagnum*-associated bacterial communities in different compartments. Rare species performed a consistent variation pattern with WBC in structures, predicted functional profiles and response to environmental factors. Additionally, they exerted large effects on the WBC co-occurrence network. Thus, our study provides new insights about the roles of rare species on *Sphagnum*-associated microbial communities.

### Changes in Bacterial Communities

Our results validated that compartment effects drove the shift of bacterial communities in *S. palustre* as indicated by alpha and beta diversities. Alpha diversity decreased from SP to Ecto and was the lowest in Endo (Figure 1). This result agrees with the view of plant-associated microbial colonization, which shows a reduction in the species diversity from rhizosphere to rhizoplane to endosphere (Edwards et al., 2015; Beckers et al., 2017). The changes of alpha diversity from SP to Ecto to Endo may closely relate to the specific tissues of *Sphagnum* comprised of chlorocytes and hyalocytes. Microbial colonization in *Sphagnum* is strictly selected by chlorocytes (Opelt et al., 2007b; Bragina et al., 2012), and reduced the bacterial diversity in *Sphagnum* compartments (Figure 1). The significant increase of community dissimilarities (beta diversity) between compartments from Ecto vs. Endo to SP vs. Ecto and further to SP vs. Endo (Figure 2C and Table 1) also confirmed plant compartment effects on bacterial communities. Each compartment is a unique and heterogeneous microenvironment (Coleman-Derr et al., 2016; Stacie et al., 2016), which selects their specific microbial communities (Beckers et al., 2017). The plant ectosphere is usually oligotrophic with frequent fluctuations in temperature, precipitation, moisture and radiation, which is challenging for microbial survival (Müller et al., 2016). Therefore microorganisms living on the surface of plants have to deal with hard conditions, which resulted in a dispersive distribution of bacterial communities in Ecto (Figure 2A). In contrast, plant cells can act as oases for microbial survival in peatlands. Usually, microorganisms with specific traits, e.g., chemotaxis, motility, and quorum sensing are recruited by the host-dependent selection or passive transport, and thus lead to the formation of a set of specific plant-associated microbial communities (Compant et al., 2010; Kardol and Wardle, 2010; Bulgarelli et al., 2012; Philippot et al., 2013; Müller et al., 2016). Unlike vascular plants, rootless *Sphagnum* mosses intercept and hold water and nutrients efficiently through non-photosynthetic cells and adjacent photosynthetic cells (Weston et al., 2015; Kostka et al., 2016), which can facilitate the capture of their endosymbiotic microorganisms.

Our results for the first time indicated that microbial taxa with various abundances accounted for different variations of microbial communities in a subalpine peatland. The richness of RT was more than 60 times higher than that of AT (Figure 1), suggesting their important contributions to microbial diversity (Lynch and Neufeld, 2015; Zhan and Macisaac, 2015). Variation patterns of microbial taxa with different abundances in alpha and beta diversities among *S. palustre* compartments (Figures 1, 2) supported the hypothesis 1 that rare species mainly contributed to bacterial community variations compared to abundant taxa.

### Co-occurrence Pattern of Communities in *Sphagnum* Compartments

The results of co-occurrence network analysis demonstrated keystone species were crucial to the whole bacterial communities and might exert large effects on other community components. Co-occurrence network can provide profound and unique insights into microbial interactions and community assembly (Barberán et al., 2012). Our results indicated that more positive interactions were observed between RT and CART (Figure 4C) than negative ones, which suggested a possible cooperation among them (Ju et al., 2014). Keystone species are characterized by high degree and low betweenness centrality based on scale-free feature (Barabási, 2009; Berry and Widder, 2014), and recognized as the initial components and affect other community components in networks. In our study, all the keystone species identified were affiliated to RT and CART, which confirmed that rare species might play a critical role in community co-occurrence network. Among keystone species, *Candidatus Nitrotoga* are cold-adapted nitrite-oxidizing bacteria, which involved in nitrogen cycling (Alawi et al., 2007). *Spirochaeta* are anaerobic bacteria, and some members are capable of cellulose-derivative degradation (glucose and cellobiose) and methane production in peat soils (Schmidt et al., 2015; Juottonen et al., 2017). These keystone species may benefit *Sphagnum* development under extreme conditions and mediate the recycle of macroelements (C, N) (Bragina et al., 2014) and sustain ecosystem network (Layeghifard et al., 2017). Therefore, rare species may be important in supporting peatland ecosystem functioning.

To be noted, rare species widely spread in all modules, which may affect the functioning of host-associated environments (Jousset et al., 2017). For example, nitrogen-fixing and methane-oxidizing microbes play essential roles in nitrogen supply for *Sphagnum* (Larmola et al., 2014; Vile et al., 2014) and decomposition of organic matter (Chen and Murrell, 2010). In the predicted functional communities, the variations in relative abundance of genes encoding nitrogenase and methane monooxygenase in RT and CART were consistent with those in WBC among compartments (Figure 5). Therefore, we surmised that rare species were important in the co-occurrence network and specific functions of bacterial communities, and the hypothesis 2 was supported. Although network analysis can give us some insight of microbial interactions, it only shows a statistical correlation among microbial groups and does not directly prove microbial interactions. Combination of microscopy-based or co-culture experiments with co-occurrence analysis will provide more direct evidences of microbial interactions.

### Environmental Factors Shaping Bacterial Communities

Environmental conditions strongly impact the structure of microbial communities (Bahram et al., 2018). The effects of water table and total nitrogen content on bacterial communities in SP samples (Table 3) matches well with previous studies in different types of peatlands (Mishra et al., 2014; Urbanová and Bárta, 2016; Zhong et al., 2017). Interestingly we further found that RT and CART also responded to water table and total nitrogen content. In contrast, AT was sensitive to organic matter content. This indicated that microbial taxa with various abundances responded differently to environmental factors. It has been demonstrated that the content and form of organic matter affect the bioavailability and the quality of organic carbon, and further affect the priority of microbial metabolism (Stres et al., 2008). Our results further demonstrated that organic matter content mainly affect AT in WBC. Therefore, the hypothesis 3 that rare species and abundant taxa responding differently to environmental factors was supported.

Microbial groups with different abundances showed distinct environmental responses, which may result from the environmental filtering or dispersal limitation (Chen et al., 2017; Wu et al., 2017; Xue et al., 2018). Our results (Table 3) indicated RT and CART were more sensitive to environmental filtering than AT did. Usually RT and CART showed weak competition capability and low growth rate (Campbell et al., 2011; Logares et al., 2015), whereas AT can compete for more substrates and well adapt to an ecosystem via active growth and proliferation in a wide niche (Pedrós-Alió, 2006; Jousset et al., 2017; Xue et al., 2018). This suggests that the composition of microbial communities is strongly affected by environmentally induced species sorting (Roberto et al., 2018). Therefore, taxa with various abundances in bacterial communities from SP were impacted by unequal environmental filtering.

## CONCLUSION

Our results demonstrated that the structures and predicted functions of rare species largely shifted across different compartments of *S. palustre* in accordance with those of whole bacterial communities. These results expanded our knowledge about the compartment effects on bacterial communities in the peatlands by emphasizing the importance of rare species. Co-occurrence network analysis revealed that rare species may play central roles in bacterial communities. Like whole bacterial communities, rare species were also significantly shaped by water table and total nitrogen content in SP, whereas abundant taxa correlated more closely with organic matter content. Collectively, our results emphasize the important impact of rare species on bacterial communities. Rare species mainly contribute to the variations both in structures and potential functions of *Sphagnum*-associated microbial communities among compartments in subalpine peatland ecosystems.

## Data Availability Statement

The datasets generated for this study can be found in the NCBI Sequence Read Archive database (PRJNA512496).

## Author Contributions

WT conducted the experiments, analyzed the data, and drafted the manuscript. XX helped the data analyses. LM, SE, and XQ gave suggestions about the manuscript. RW helped the sample collection. HW designed the research, provided the funding, and revised the manuscript.

## Conflict of Interest

The authors declare that the research was conducted in the absence of any commercial or financial relationships that could be construed as a potential conflict of interest.

## Footnotes

1 https://www.ncbi.nlm.nih.gov/sra

2 http://folk.uio.no/ohammer/past/

3 https://www.r-project.org/

4 https://spss.en.softonic.com/

5 http://canoco5.com/

6 https://gephi.org/
