# Supplementary material for: Rare Species Shift the Structure of Bacterial Communities Across Sphagnum Compartments in a Subalpine Peatland
Source: Front Microbiol. 2020 Jan 21;10:3138. doi: 10.3389/fmicb.2019.03138 (PMC6986206; doi:10.3389/fmicb.2019.03138)
Supplement: Supplementary file 1 [file Data_Sheet_1.docx]

Rare Species Shift the Structure of Bacterial Communities across *Sphagnum* Compartments in a Subalpine Peatland

***Wen Tian^1^, Xing Xiang^1^, Liyuan Ma^2^, Stephanie Evers^3, 4^, Ruicheng Wang^1^, Xuan Qiu^1^, Hongmei Wang^1, 2, 5*^***

^1^State Key Laboratory of Biogeology and Environmental Geology, China University of Geosciences, Wuhan, China

^2^School of Environmental Studies, China University of Geosciences, Wuhan, China

^3^School of Natural Sciences and Psychology, Liverpool John Moores University, Liverpool, UK

^4^TROCARI (Tropical Catchment Research Initiative), Malaysia

^5^Laboratory of Basin Hydrology and Wetland Eco-restoration, China University of Geosciences Wuhan, China

***Correspondence:**

Hongmei Wang

hmwang@cug.edu.cn; [wanghmei04@163.com](mailto:wanghmei04@163.com)


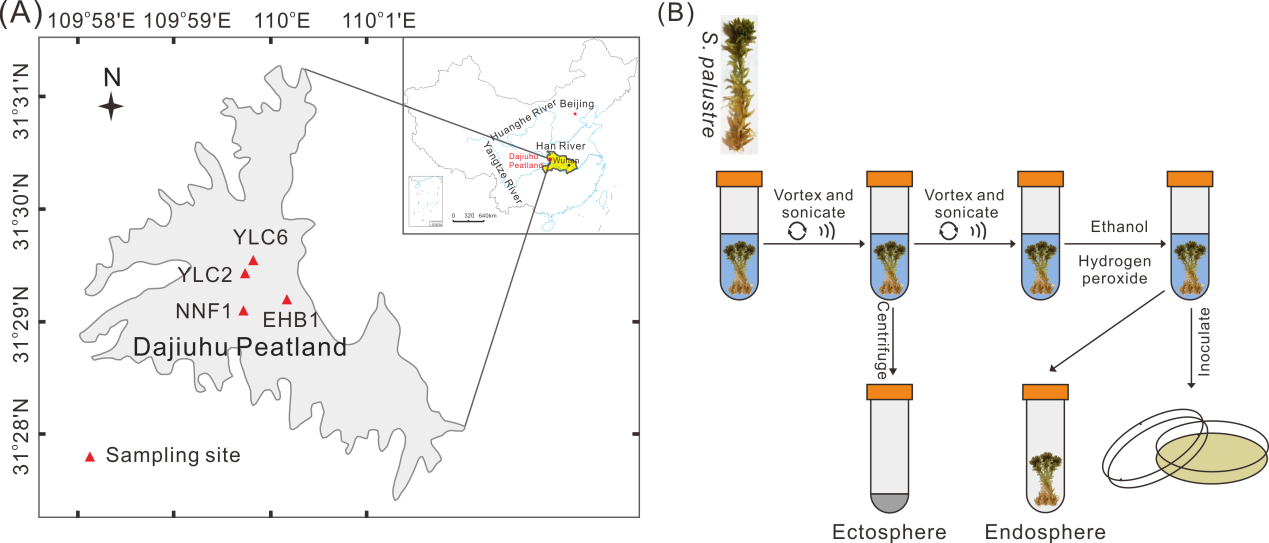


**Supplementary Figure S1.** Location of the study area and sampling sites **(A)** and processes of *S. palustre* **(B)** modified from [Edwards et al. (2015](#_ENREF_1)).

References:

Edwards J, Johnson C, Santos-Medellín C, Lurie E, Podishetty NK, Bhatnagar S, Eisen JA, Sundaresan V (2015) Structure, variation, and assembly of the root-associated microbiomes of rice. Proc. Natl. Acad. Sci. USA 112: E911-E920. <https://doi.org/10.1073/pnas.1414592112>


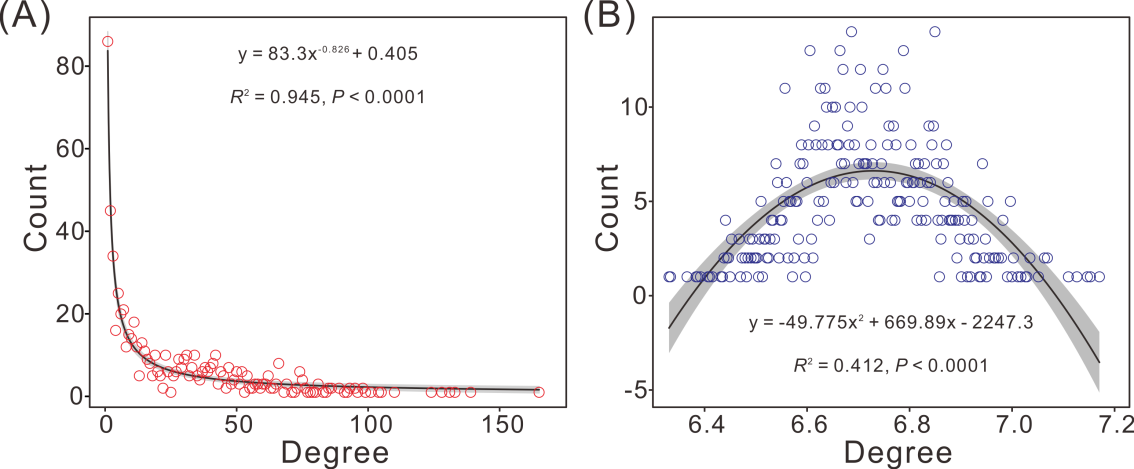


**Supplementary Figure S2.** The distributions of degrees in the real network **(A)** and Erdös-Rényi random network **(B)** of *S. palustre* bacterial communities.


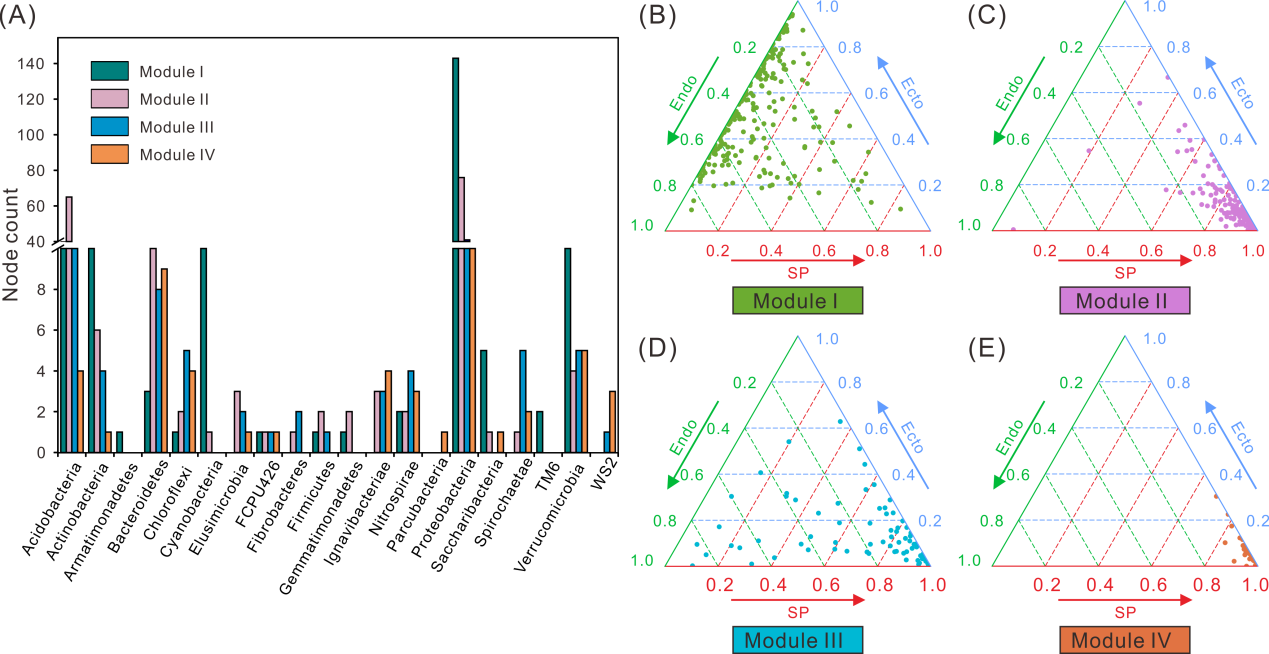


**Supplementary Figure S3.** Taxonomic compositions **(A)** and ternary plots **(B)** of each module in the co-occurrence network of bacterial communities in the Dajiuhu Peatland. Each circle represents a single OTU. SP, *S. palustre* peat; Ecto, *S. palustre* ectosphere; Endo, *S. palustre* endosphere.


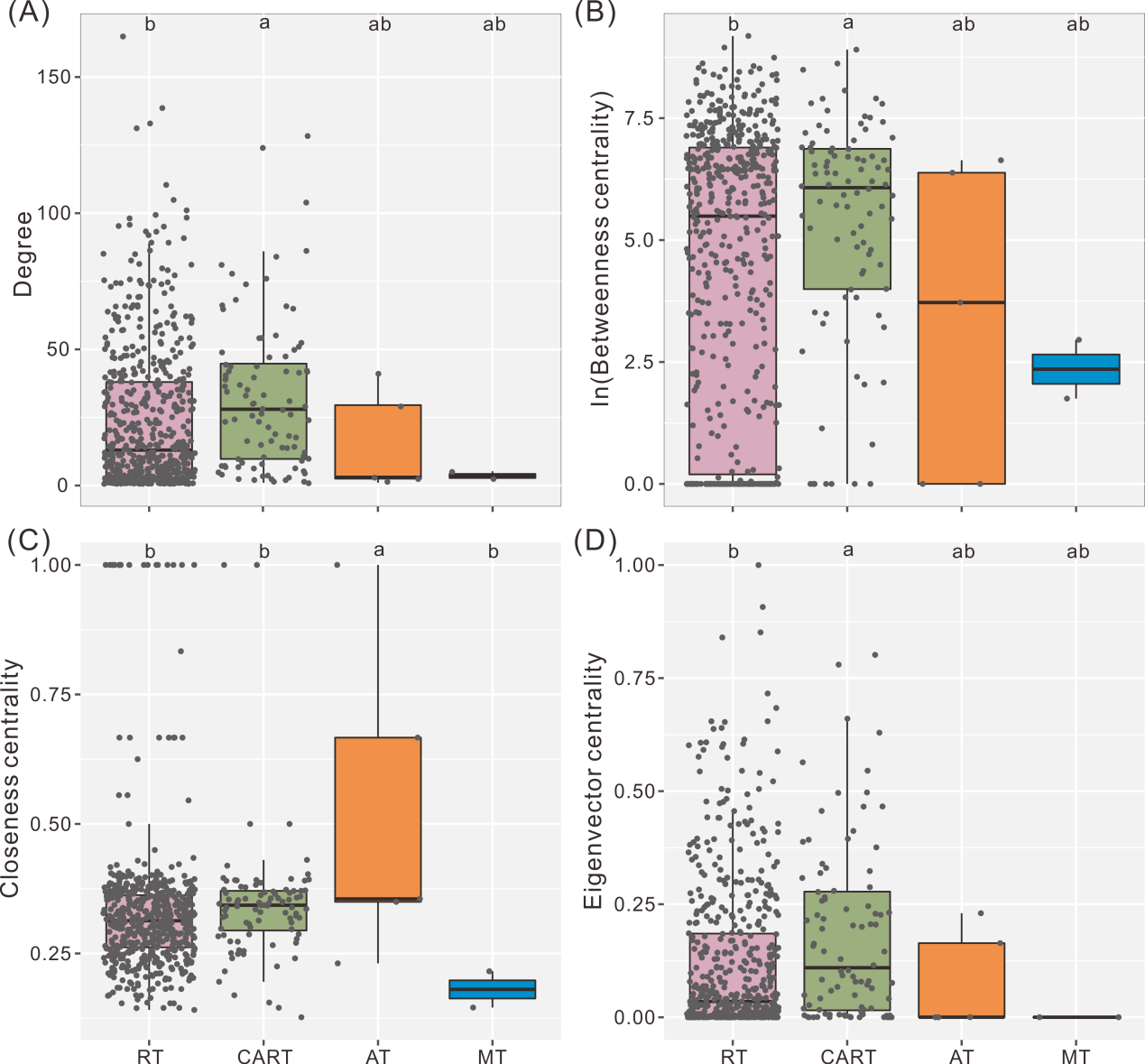


**Supplementary Figure S4.** Comparison of node-level topological features in microbial groups with different relative abundances. Each box plot represents the maximum and minimum, median, 75^th^ and 25^th^ quartile values. Lowercases letters indicate the significant level (α = 0.05) by ANOVA with Tukey post hoc comparisons. RT, rare taxa; CART, conditionally abundant and rare taxa; AT, abundant taxa; MT, moderate taxa.


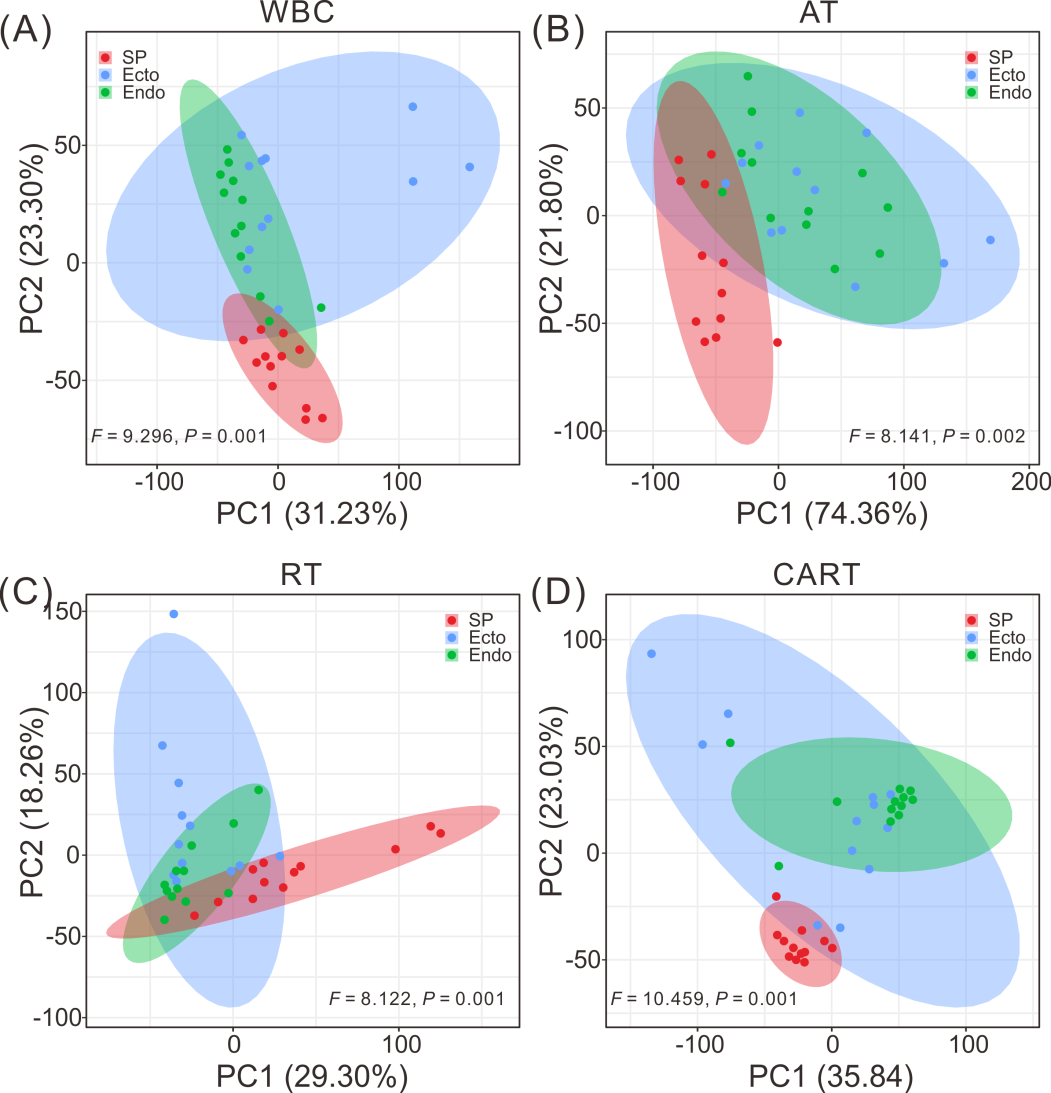


**Supplementary Figure S5.** Principal component analysis (PCA) showing community variations based on the predicted functions across *S. palustre* compartments in WBC **(A)**, AT **(B)**, RT **(C)** and CART **(D)**. Dissimilarities among the samples from different compartments are examined via PERMANOVA test based on Euclidean distance of the relative abundance in all Kyoto Encyclopedia of Genes and Genomes (KEGG) Orthologs (KOs). WBC, whole bacterial communities; AT, abundant taxa; RT, rare taxa; CART, conditionally abundant and rare taxa.

**Table S1.** Definition of abundant and rare OTUs and their relative abundances in bacterial communities in the Dajiuhu Peatland.

| **Category** | **Subcategory** | **OTU numbers** | **Sequence percentage (%)** | **Dissimilarity contribution (%)** |
| --- | --- | --- | --- | --- |
| WBC | WBC | 2,848 | 100 | 100 |
| AT | AAT | 0 | 0 | 0 |
|  | CAT | 13 | 9.01 ±3.88 | 5.87 |
| RT | ART | 397 | 0.12 ±0.09 | 0.15 |
|  | CRT | 2,309 | 39.08 ±12.40 | 40.63 |
| MT | MT | 3 | 0.65 ±0.45 | 0.37 |
| CART | CART | 126 | 51.13 ± 14.56 | 52.98 |

*WBC, whole bacterial communities; AT, abundant taxa including always abundant taxa (AAT) and conditionally abundant taxa (CAT); AAT are defined as the OTUs with a relative abundance > 1% in all samples; CAT are defined as the OTUs with a relative abundance ≥ 1% in some samples and always ≥ 0.01% in all samples; RT, rare taxa including always rare taxa (ART) and conditionally rare taxa (CRT). ART are defined as the OTUs with a relative abundance* *< 0.01% in all samples; CRT are defined as the OTUs with a relative abundance < 0.01% in some samples but never abundant (≥ 1%) in any samples; MT, moderate taxa are defined as the OTUs with a relative abundance between 0.01% and 1% in all samples; CART, conditionally abundant and rare taxa are defined as the OTUs with a relative abundance varying from rare (< 0.01%) to abundant (≥ 1%).*

**Table S2.** Analysis of variance (ANOVA) in alpha diversity of bacterial communities across *S. palustre* compartments and sampling sites.

|  | **WBC** | | **AT** | | **RT** | | **CART** | |
| --- | --- | --- | --- | --- | --- | --- | --- | --- |
|  | ***F*** | ***P*** | ***F*** | ***P*** | ***F*** | ***P*** | ***F*** | ***P*** |
| Compartment | | | | | | | | |
| Observed species | 10.941 | **0.000** | -- | -- | 10.025 | **0.000** | 11.133 | **0.000** |
| Chao1 | 11.670 | **0.000** | -- | -- | 11.314 | **0.000** | 1.168 | 0.323 |
| ACE | 11.251 | **0.000** | -- | -- | 10.598 | **0.000** | 2.810 | 0.075 |
| Simpson | 11.157 | **0.000** | 2.503 | 0.097 | 5.604 | **0.008** | 16.584 | **0.000** |
| Pielou | 20.601 | **0.000** | 2.925 | 0.068 | 3.379 | **0.046** | 25.976 | **0.000** |
| Shannon | 21.482 | **0.000** | 2.925 | 0.068 | 5.607 | **0.008** | 32.097 | **0.000** |
| Sampling site | | | | | | | | |
| Observed species | 0.639 | 0.596 | -- | -- | 0.762 | 0.524 | 1.547 | 0.221 |
| Chao1 | 0.630 | 0.601 | -- | -- | 0.614 | 0.611 | 3.846 | **0.019** |
| ACE | 0.578 | 0.634 | -- | -- | 0.578 | 0.633 | 1.832 | 0.161 |
| Simpson | 2.786 | 0.057 | 2.270 | 0.099 | 2.215 | 0.105 | 1.864 | 0.156 |
| Pielou | 1.987 | 0.136 | 2.501 | 0.077 | 2.491 | 0.078 | 1.788 | 0.169 |
| Shannon | 1.859 | 0.156 | 2.501 | 0.077 | 1.522 | 0.228 | 1.466 | 0.242 |

*The F and P values of Observed species, Chao1 and ACE indices cannot be calculated in AT.* *The abbreviations refer to those in Table S1. The differences of bacterial communities with various abundances in alpha diversity are tested by ANOVA. P values < 0.05 are in bold.*

**Table S3.** The number of OTUs and sequences within each taxonomic group at the phylum level.

|  | **WBC** | | **AT** | | **RT** | | **CART** | |
| --- | --- | --- | --- | --- | --- | --- | --- | --- |
|  | **OTU number** | **Sequence** | **OTU number** | **Sequence** | **OTU number** | **Sequence** | **OTU number** | **Sequence** |
| Acidobacteria | 304 | 161,876 | 0 | 0 | 279 | 87,935 | 24 | 72,709 |
| Actinobacteria | 187 | 59,466 | 3 | 16,259 | 176 | 28,870 | 7 | 10,835 |
| Aminicenantes | 2 | 119 | 0 | 0 | 2 | 119 | 0 | 0 |
| Armatimonadetes | 27 | 1,272 | 0 | 0 | 27 | 1,272 | 0 | 0 |
| Bacteroidetes | 177 | 21,856 | 0 | 0 | 173 | 15,439 | 4 | 6,417 |
| Chlamydiae | 9 | 347 | 0 | 0 | 9 | 347 | 0 | 0 |
| Chlorobi | 5 | 293 | 0 | 0 | 5 | 293 | 0 | 0 |
| Chloroflexi | 34 | 14,474 | 0 | 0 | 30 | 3,365 | 4 | 11,109 |
| CPR2 | 2 | 29 | 0 | 0 | 2 | 29 | 0 | 0 |
| Cyanobacteria | 81 | 214,788 | 0 | 0 | 74 | 8,493 | 7 | 206,289 |
| Elusimicrobia | 44 | 3,131 | 0 | 0 | 43 | 2,455 | 1 | 676 |
| FCPU426 | 12 | 5 | 0 | 0 | 12 | 1,359 | 0 | 0 |
| Fibrobacteres | 10 | 1,359 | 0 | 0 | 10 | 761 | 0 | 0 |
| Firmicutes | 63 | 761 | 0 | 0 | 60 | 2,967 | 3 | 6,606 |
| Gemmatimonadetes | 10 | 1,801 | 0 | 0 | 10 | 1,801 | 0 | 0 |
| Ignavibacteriae | 19 | 7,263 | 0 | 0 | 15 | 2,692 | 4 | 4,571 |
| KSB3 | 1 | 8 | 0 | 0 | 1 | 8 | 0 | 0 |
| Nitrospirae | 26 | 9,432 | 0 | 0 | 24 | 4,307 | 2 | 5,125 |
| Omnitrophica | 1 | 3 | 0 | 0 | 1 | 3 | 0 | 0 |
| Parcubacteria | 9 | 473 | 0 | 0 | 9 | 473 | 0 | 0 |
| Peregrinibacteria | 1 | 14 | 0 | 0 | 1 | 14 | 0 | 0 |
| Planctomycetes | 11 | 243 | 0 | 0 | 11 | 243 | 0 | 0 |
| Proteobacteria |  |  |  |  |  |  |  |  |
| Alphaproteobacteria | 589 | 239,624 | 8 | 58,500 | 552 | 97,719 | 28 | 81,789 |
| Betaproteobacteria | 167 | 45,974 | 0 | 0 | 158 | 30,155 | 9 | 15,819 |
| Deltaproteobacteria | 307 | 32,556 | 0 | 0 | 300 | 24,939 | 7 | 7,617 |
| Epsilonproteobacteria | 1 | 5 | 0 | 0 | 1 | 5 | 0 | 0 |
| Gammaproteobacteria | 375 | 107,722 | 1 | 6,890 | 355 | 35,202 | 19 | 65,630 |
| RBG-1 | 1 | 314 | 0 | 0 | 1 | 314 | 0 | 0 |
| Saccharibacteria | 87 | 6,752 | 0 | 0 | 87 | 6,752 | 0 | 0 |
| Spirochaetae | 31 | 5,151 | 0 | 0 | 29 | 2,872 | 2 | 2,279 |
| SR1 | 1 | 2 | 0 | 0 | 1 | 2 | 0 | 0 |
| TM6 | 89 | 4,930 | 0 | 0 | 87 | 3,370 | 2 | 1,560 |
| Unclassified Proteobacteria | 60 | 18,017 | 1 | 6,428 | 57 | 5,981 | 2 | 5,608 |
| Verrucomicrobia | 99 | 12,909 | 0 | 0 | 99 | 12,909 | 0 | 0 |
| WS2 | 6 | 1,521 | 0 | 0 | 5 | 881 | 1 | 640 |
| Total | 2,848 | 984,508 | 13 | 88,077 | 2,706 | 384,346 | 126 | 505,278 |

*Abbreviations are the same as indicated by* ***Table S1****.*

**Table S4.** Keystone species in the co-occurrence network of bacterial communities in the Dajiuhu Peatland.

| **OTU_ID** | **Category** | **Modularity**  **class** | **Betweenness centrality** | **Degree** | **Taxonomy** |
| --- | --- | --- | --- | --- | --- |
| OTU_237 | CART | II | 3186 | 104 | k_Bacteria;p_Ignavibacteriae;c_Ignavibacteria;o_Ignavibacteriales;f_BSV26 |
| OTU _1612 | RT | II | 2543 | 101 | k_Bacteria;p_Spirochaetae;c_Spirochaetes;o_Spirochaetales;f_Spirochaetaceae;g_*Spirochaeta* |
| OTU _1746 | RT | II | 3391 | 105 | k_Bacteria;p_Proteobacteria;c_Alphaproteobacteria;o_Rhizobiales;f_Xanthobacteraceae |
| OTU _455 | RT | II | 4671 | 139 | k_Bacteria;p_Proteobacteria;c_Betaproteobacteria;o_Nitrosomonadales;f_Gallionellaceae;g_*Candidatus Nitrotoga* |
| OTU _458 | RT | II | 4419 | 133 | k_Bacteria;p_Acidobacteria;c_Acidobacteria;o_Acidobacteriales;f_Acidobacteriaceae Subgroup 1 |

*Nodes with high degree (> 100) and low betweenness centrality value (< 5,000) are identified as keystone species in co-occurrence networks.*

*Abbreviations are the same as those described in* ***Table S1****.*

**Table S5.** Dissimilarity test showing differences in microbial functional groups across *S. palustre* compartments and sampling sites.

|  | **PERMANOVA** | | | | | | | |
| --- | --- | --- | --- | --- | --- | --- | --- | --- |
|  | **WBC** | | **AT** | | **RT** | | **CART** | |
|  | ***F*** | ***P*** | ***F*** | ***P*** | ***F*** | ***P*** | ***F*** | ***P*** |
| Compartment |  |  |  |  |  |  |  |  |
| SP vs. Ecto | 10.233 | **0.002** | 14.330 | **0.002** | 9.183 | **0.002** | 8.164 | **0.002** |
| SP vs. Endo | 13.024 | **0.002** | 13.194 | **0.002** | 9.751 | **0.002** | 23.240 | **0.002** |
| Ecto vs. Endo | 3.221 | **0.013** | 0.704 | 0.401 | 1.826 | 0.125 | 3.176 | **0.010** |
| Sampling site |  |  |  |  |  |  |  |  |
| E1 vs. N1 | 0.633 | 0.658 | 10.189 | **0.002** | 1.483 | 0.188 | 0.643 | 0.784 |
| E1 vs. Y2 | 1.022 | 0.437 | 17.353 | **0.002** | 1.927 | 0.139 | 0.527 | 0.784 |
| E1 vs. Y6 | 3.208 | 0.120 | 12.573 | **0.002** | 6.996 | **0.006** | 1.718 | 0.268 |
| N1 vs. Y2 | 2.036 | 0.165 | 4.346 | 0.074 | 2.808 | 0.069 | 0.536 | 0.784 |
| N1 vs. Y6 | 4.613 | 0.084 | 0.285 | 0.751 | 6.953 | **0.006** | 3.228 | 0.261 |
| Y2 vs. Y6 | 2.669 | 0.158 | 3.634 | 0.080 | 4.468 | 0.018 | 2.114 | 0.261 |

*PERMANOVA, permutational multivariate analysis of variance; SP, S. palustre peat; Ecto, S. palustre ectosphere; Endo, S. palustre endosphere; E1, the first site of Erhaoba; N1, the first site of Niangniangfen; Y2, the second site of Yangluchang; Y6, the sixth site of Yangluchang. Dissimilarities of predicted functional communities were calculated based on Euclidean matrix of the relative abundance of KOs. P values are corrected by false discovery rate in BH method. Bold font represents significant value (α = 0.05). Abbreviations are the same as those in* ***Table S1****.*
